# Evaluation of seasonal variations for the seasonal pattern assessment in mood disorder patients and healthy controls

**DOI:** 10.1186/s12888-025-06916-y

**Published:** 2025-05-07

**Authors:** Chiao-Erh Chang, Hsi-Chung Chen, I.-Ming Chen, Cheng-Dien Hsu, Chih-Ming Liu, Chun-Hsin Chen, Tsung-Yang Wang, Wen-Yin Chen, Shiau-Shian Huang, Yen-Chih Chen, Po-Hsiu Kuo

**Affiliations:** 1 Department of Public Health & Institute of Epidemiology and Preventive Medicine, College of Public Health, National Taiwan University, Taipei, Taiwan; 2 Department of Psychiatry, National Taiwan University Hospital, Taipei, Taiwan; 3 Center of Sleep Disorders, National Taiwan University Hospital, Taipei, Taiwan; 4 Department of Psychiatry, College of Medicine, National Taiwan University, Taipei, Taiwan; 5 Department of Psychiatry, Adventist Hospital, Taipei, Taiwan; 6 Department of Psychiatry, Wan Fang Hospital, Taipei Medical University, Taipei, Taiwan; 7 Department of Psychiatry, School of Medicine, College of Medicine, Taipei Medical University, Taipei, Taiwan; 8 Taipei City Psychiatric Center, Taipei City Hospital, Songde Branch, Taipei, Taiwan; 9 School of Medicine, College of Medicine, Fu Jen Catholic University, New Taipei City, Taiwan; 10 Department of Medical Education, Taipei Veterans General Hospital, Taipei, Taiwan; 11 College of Medicine, National Yang Ming Chiao Tung University, Taipei, Taiwan; 12 School of Public Health, National Defense Medical Center, Taipei, Taiwan; 13 Nankung Psychiatric Hospital, Keelung, Taiwan; 14 Department of Physical Medicine and Rehabilitation, National Taiwan University Hospital and National Taiwan University College of Medicine, Taipei, Taiwan; 15 Psychiatric Research Center, Wan Fang Hospital, Taipei Medical University, Taipei, Taiwan

**Keywords:** Seasonal affective disorder, Bipolar disorder, Major depressive disorder, Seasonal pattern, Seasonal pattern assessment questionnaire

## Abstract

**Background:**

Seasonal disturbances were common in mood disorders patients. The global seasonality score (GSS), derived from the Seasonal Pattern Assessment Questionnaire (SPAQ), was widely used to assess seasonality and related symptoms. This study aimed to establish the structure of the Chinese version of SPAQ. We examined the stability of seasonal pattern assessment across four seasons when administering SPAQ. The prevalence of seasonal affective disorder was estimated using SPAQ criteria.

**Methods:**

We recruited 596 mood disorder patients and 138 healthy controls (HC), with 121 patients and 37 HC followed up over four seasons. An exploratory factor analysis examined the GSS factor structure. We evaluated correlations between GSS symptom dimensions and “the degree of problems due to seasonal changes” and used intraclass correlation coefficient reliability (ICCR) to assess the consistency of symptom dimensions across seasons.

**Results:**

Approximately a quarter of mood disorder patients met the criteria for seasonal affective disorder. The Chinese SPAQ revealed a two-factor structure: psychological and food-related symptoms among patients. The GSS showed a significant correlation (*r* = 0.64) with the degree of problems due to seasonal changes in mood disorder patients, while energy level and sleep significantly correlated with GSS (*r* > 0.75) in HC. Reporting reliability (ICCR > 0.4) was acceptable for GSS and mood/energy levels in patients across seasons.

**Conclusions:**

Seasonal variations were observed in reporting the symptom dimensions of the seasonal pattern assessment, while the GSS remained relatively stable in both mood disorder patients and HC. SPAQ is a useful tool for measuring seasonality, irrespective of the season of administration.

**Supplementary Information:**

The online version contains supplementary material available at 10.1186/s12888-025-06916-y.

## Introduction

Mood and behavioral changes that vary with the seasons are observed in humans [1]. Some individuals with mood disorders, such as bipolar disorder (BD) and major depressive disorder (MDD), have episodic seasonal patterns in their mood, sleep, and behaviors [2–4]. This seasonality effect in mood disorders patients has been linked to dysregulated biological rhythms [3, 5], with significant seasonality being a core feature of seasonal affective disorder (SAD), which is often characterized by recurrent depressive symptoms during the winter month every year [6]. Seasonality affects various physiological processes, such as body temperature regulation, metabolism, sleep patterns, autonomic function, neurotransmission, and hormonal response to stimuli, which could potentially impact an individual’s susceptibility to different diseases and health conditions [7]. According to previous research, mood disorders patients with SAD experienced greater health risks, weight gain, and sleepiness during depressive episodes compared to those without seasonal patterns [8]. Seasonality has also been linked to worse physical conditions, including hypertension, diabetes, high cholesterol levels, and increased body mass index [8, 9].

In the mid-latitude Asian countries, data on the prevalence of SAD remain limited. In Japan, the overall prevalence of SAD has been reported to range from 1 to 3% [10]. However, the proportion of SAD among individuals with mood disorders in Japan remain unknown. In South Korea, 17.5% of patients with BD have been screened and identified as having SAD, while approximately 10% of those with MDD also met the criteria [11]. The Diagnostic and Statistical Manual of Mental Disorders, Fifth Edition (DSM-5), defines the episodic seasonal pattern as seasonal specifiers of mood episodes that recur during a particular season of the year, with seasonal episodes outnumbering non-seasonal ones [12]. However, many patients with mood disorders exhibit seasonal patterns that may not be as severe as SAD defined by DSM-5, but are still showing significant seasonal disturbance fluctuation. On this broader spectrum, it is estimated that approximately one-quarter of European patients diagnosed with BD exhibited seasonal patterns in their episodes, while the prevalence among those with MDD ranges from 10 to 20% [3, 13, 14]. There is a relative scarcity of information regarding the prevalence of seasonal patterns among mood disorder patients in Asian populations, including Taiwan, which is located at a latitude of approximately 23.5°N within subtropical regions. A recent study conducted in a region of China between latitudes 28° and 32°N reported that around 25% of BD patients and 9.9% of those with anxiety and depressive disorders met the criteria for SAD [15]. Another earlier study from northern India (27°–29°N) found that 5.68% of patients diagnosed with affective disorders based on DSM-III criteria had SAD [16]. However, it remains unclear whether similar patterns are observed in Taiwan, where seasonal changes are less pronounce, yet may still influence mood-related symptoms in susceptible individuals.

The magnitude of seasonal patterns can be evaluated using symptom dimensions affected by seasons [3]. The Seasonal Pattern Assessment Questionnaire (SPAQ) is a commonly used tool to assess seasonality in mood, symptoms, and behaviors, and has long been used to screen for SAD and subsyndromal-SAD (S-SAD) [4, 17], with the global seasonality score (GSS) derived from the SPAQ. Using this assessment can provide more detailed information about symptoms than simply diagnosing SAD. The GSS has demonstrated good internal consistency and validity in previous research, confirming its reliability as a measure of seasonality in mood and behavior [18–20]. Some studies have also reported good test–retest reliability for the GSS [20, 21].

However, emotional variability has been found to be influenced by subjective awareness, as noted by Waston [22]. In a previous study conducted in Norway, it was observed that individuals’ self-reported level of seasonality varied depending on the time of year when they completed the questionnaire [23]. Specifically, participants tended to report higher levels of seasonality during the winter months compared to autumn [23] The impact of the season in which the SPAQ is administered on the GSS is not clear. This uncertainty may lead to inaccuracies in assessing the prevalence of SAD and understanding how patients perceive seasonal changes at distinct time points in a year. Therefore, the present study aimed to examine the seasonal pattern and mood-related symptoms in Taiwanese mood disorder patients. Specifically, our objectives were to (i) establish the factor structure of the Chinese version of SPAQ, (ii) assess the stability of self-report seasonality through four-season follow-ups using SPAQ, and (iii) estimate the prevalence of SAD and Subsyndromal-SAD among mood disorder patients using SPAQ criteria.

## Materials and methods

### Subject recruitments

Participants were recruited between 2017 to 2022 and were followed for at least one year to ensure data collection across all four seasons, with initial assessments conducted at various times throughout the year. Patients diagnosed with mood disorder, including BD and MDD, according to the DSM-5 criteria were referred by psychiatrists from several hospitals in Taipei. Individuals who had a history of intellectual disability, schizophrenia, schizoaffective disorder, or substance-induced secondary mood disorder were excluded from the study. Healthy controls (HC) were recruited from the same community and were screened by well-trained interviewers using the modified Chinese version of the Schedule for Affective Disorders and Schizophrenia-Lifetime (SADS-L). Those with any history of BD and MDD, or any of the aforementioned conditions were also excluded. Both mood disorder patients and the HC participants were between 20 and 70 years of age. A total of 735 subjects (597 mood disorder and 138 HC) participated in the present study, and 158 subjects (121 mood disorder patients and 37 HC) had at least two follow-up measurements. All questionnaires were administered by well-trained interviewers.

### The seasonal pattern assessment

We used the Chinese SPAQ version to evaluate the presence of mood swings associated with seasonality. Subjects were required to complete the Chinese SPAQ at least once during the baseline and each follow-up season, depending on their involvement in the one-year follow-up periods. The Chinese SPAQ was adapted from the original version developed by Rosenthal et al. [17]. The questionnaire consists of five sections. The first section collects demographic information, including gender and region of residence. The second section evaluates seasonal changes in six symptom dimensions: sleep, social activity, mood, weight, appetite, and energy level. The degree of seasonal change is rated on a scale of 0 to 4, representing no change, mild, moderate, marked, and extreme changes, respectively. The third section explores whether subjects consider these seasonal changes in symptom dimensions to be problematic and, if so, to what extent. The score ranged from 1 (mild) to 5 (disabling). The fourth section investigates the patterns of seasonal change (the ‘season items’), where subjects indicate which season they experience more severe depressive or manic symptoms. Finally, the fifth section examines the patterns of seasonal change at the monthly level, with subjects indicating which month they perceive as worse (for depression) or better (for mania) in terms of mood, sleep, energy, and fatigue.

The GSS was calculated as the sum of six symptom dimensions, ranging from 0 (indicating no seasonality) to 24 (extreme seasonality). The criterion for defining SAD was a GSS of ≥ 11, along with mood disorder patients experiencing problems of greater than moderate levels due to seasonal changes [4]. In addition, S-SAD was defined as (1) a GSS ≥ 11 with mood disorder patients experiencing problems of mild levels of problems due to seasonal changes, or (2) a GSS of 9 or 10 with mood disorder patients experiencing problems of greater than mild levels of problems due to seasonal changes [4]. The SPAQ classifications have been reported with good specificity (94%) but low sensitivity (44%) and the GSS symptom severity have proven to have good internal consistency (alpha = 0.82—0.85) in both the general population and clinical mood disorder patients [18, 24]. The SPAQ can also aid in classifying different seasonal types of patients with SAD [18]. The seasonal types were determined based on the ‘month items’ of seasonal change. Winter-type patients exhibited episode symptoms between December and February, with no occurrence in other months. On the contrary, summer-type patients experienced symptoms between June and August. Spring-type and autumn-type were defined as symptoms occurring between March and May, and between September and November, respectively.

### Statistical analysis

We used the student’s *t*-test and Pearson’s χ2 test to examine the differences in demographics and clinical characteristics between mood disorder patients and the HC group. In order to examine the factor structure of the GSS in the present study, we conducted an exploratory factor analysis (EFA) with Promax rotation separately for the mood disorder and HC groups. Model fit was assessed using the root mean square error of approximation (RMSEA, < 0.06) and the root mean square residual (RMSR, < 0.08) criteria [25]. The Spearman correlation coefficient was utilized to estimate the correlations among the total GSS, the six symptom dimensions, and the levels of problems due to seasonal changes. To assess the reliability of measurements collected across the four seasons for the six symptom dimensions, GSS, and the patterns of seasonal change (‘season items’), we used the intraclass correlation coefficient reliability (ICCR). The ICCR is particularly suitable for evaluating reliability in studies with repeated measurements, even when the number of measurements per subject varies. ICCR values less than 0.5 indicate poor reliability, values between 0.5 and 0.75 indicate moderate reliability, values between 0.75 and 0.9 indicate good reliability, and values greater than 0.9 indicate excellent reliability [26]. We further used generalized estimating equations with an exchangeable correlation structure to account for repeated measures within individuals. The overall effect of the month on SAD prevalence among individuals with mood disorder was assessed using a Wald chi-square test. A two-sided *p*-value of < 0.05 was considered statistically significant. All statistical analyses were performed using SAS software version 9.4 (SAS Institute, Cary, NC, USA). The EFA was conducted using Mplus software version 1.8.7. Data visualization was performed using R software version 4.0.2.

## Results

### Demographic, seasonal characteristics, and SAD proportion

The demographic and clinical characteristics are presented in Table 1. Mood disorder patients were observed to be older (mean age: 44.11 years vs. 37.84 years old, *p* < 0.001) and demonstrated greater severity of seasonality (GSS: 7.59 v. 3.79, *p* < 0.001) compared to the HC group. In total, approximately one-quarter of mood disorder patients fulfilled the criteria for SAD, while 13.74% met the criteria for S-SAD. During the follow-up periods spanning four seasons, the mean follow-up times were 3.66 times for mood disorder patients and 3.59 times for the HC group, respectively. Among mood disorder patients with multiple measurements, 36% and 18% of patients met the criteria for SAD or S-SAD, respectively. Specifically, 50% of them met the SAD criterion once, 29.55% met it twice, 9.09% met it three times, and 11.36% met it four times (Table S1). The distribution of completed SPAQ across each season was as follows: 294 in spring, 287 in summer, 268 in fall, and 303 in winter (Table S1).

**Table 1:** Demographic and clinical characteristics of patients with mood disorders and healthy controls (*n* = 734)

Variables	Mood disorder patients (*n* = 596)^a^		Healthy controls (*n* = 138)^b^	
	Once (*n* = 475)	Multiple (*n* = 121)	Once (*n* = 101)	Multiple (*n* = 37)
Age, mean (standard deviation)	43.76 (13.75)	45.47 (13.40)	37.65 (13.21)	38.38 (13.13)
Female gender, *N* (%)	329 (69.26)	78 (64.46)	69 (68.32)	23 (62.16)
Diagnosis				
Bipolar disorder, *N* (%)	228 (48.00)	70 (57.85)	-	-
Major depressive disorder, *N* (%)	247 (52.00)	51 (42.15)	-	-
Average times of repeat measurement, mean (standard deviation)	-	3.66 (1.03)	-	3.59 (1.07)
GSS, mean (standard deviation)	7.49 (5.63)	7.95 (5.04)	3.66 (3.20)	4.14 (3.25)
SAD^c^, *N* (%)	106 (22.32)	44 (36.36)	-	-
S-SAD^d^, *N* (%)	60 (12.63)	22 (18.18)	-	-

*GSS* Global seasonality score, *SAD* Seasonal affective disorder, *S-SAD* Subsyndromal- Seasonal affective disorder

^a^A total of 918 records in 596 mood disorder patients

^b^A total of 234 records in 138 healthy participants

^c^Patients with multiple seasonal pattern assessments who met the SAD criteria at least once

^d^Patients with multiple seasonal pattern assessments who met the S-SAD criteria at least once

We further investigated the seasonal patterns observed in patients with SAD based on their responses collected across different seasons (refer to Table 2). In terms of depressive episodes, 41.67% of MDD patients displayed a winter pattern, 22.22% exhibited a summer pattern, and 2.78% reported experiencing both patterns. Among patients diagnosed with bipolar I disorder (BD-I) and bipolar II disorder (BD-II), 51.32% and 59.26%, respectively, demonstrated a winter pattern of depression, while 26.32% and 14.81%, respectively, showed a summer pattern of depression. Additionally, 18.42% of BD-I patients and 22.22% of BD-II patients reported experiencing seasonal patterns in two other seasons. Moving on to manic episodes, more than half of BD-I and BD-II patients exhibited a summer pattern, while 8.62% to 9.09% were classified as having a spring pattern. Furthermore, almost a quarter of BD-I and BD-II patients reported experiencing a seasonal pattern in two other seasons.

**Table 2:** The seasonal pattern types in mood disorder patients with seasonal affective disorder

	BD-I	BD-II	MDD
Depression, *n* (%)	*n* = 76	*n* = 27	*n* = 72
Winter pattern	39 (51.32)	16 (59.26)	30 (41.67)
Summer pattern	20 (26.32)	4 (14.81)	16 (22.22)
Spring and autumn	14 (18.42)	6 (22.22)	24 (33.33)
(Hypo) mania, *n* (%)	*n* = 58	*n* = 13	
Spring pattern	5 (8.62)	2 (9.09)	-
Summer pattern	29 (50.00)	7 (63.64)	-
Autumn and Winter	18 (31.03)	3 (27.27)	

In the case of participants with multiple measurements, the season type was determined based on whether each measurement met the criteria for a seasonal affective disorder diagnosis

*MDD* Major depressive disorder, *BD-I* Bipolar I disorder, *BD-II* Bipolar II disorder

### The structure of the global seasonality score

A two-factor structure of the GSS was identified among mood disorder patients, as presented in Table 3. Factor 1 consisted of sleep, social activity, mood, and energy level dimensions, which reflected the psychological aspect. Factor 2 was associated with the food-related dimensions of weight and appetite. The goodness-of-fit statistics indicated a satisfactory model fit, with an RMSEA of 0.06, RMSR of 0.01, and factor correlation of 0.64. In contrast, the HC group exhibited a unifactorial structure, encompassing all six dimensions, with most of the model fit indices within acceptable ranges (TLI = 0.91, RMSEA = 0.13, RMSR = 0.06, BIC = –15.6). The slightly higher RMSEA may result from smaller sample size in the HC group with one factor solution. All six items loaded strongly and exclusively on a single factor (loadings = 0.58–0.80), without cross-loadings. These results support the overall adequacy and interpretability of the model in our samples.

**Table 3:** Factor loadings of exploratory factor analysis for global seasonality score

	MD		HC
	Factor 1	Factor 2	Factor 1
Rotated factor loading			
Sleep	**0.64**	0.14	**0.75**
Social activity	**0.75**	0.07	**0.79**
Mood	**0.91**	-0.09	**0.77**
Weight	-0.04	**0.91**	**0.72**
Appetite	0.18	**0.72**	**0.73**
Energy level	**0.80**	0.13	**0.85**
Eigenvalue	3.93	0.82	3.89
RMSEA	0.06		0.13
RMSR	0.01		0.07
Factor correlations	0.63		-

Bold presents the factor loading higher than 0.6

*RMSEA* Root Mean Square Error of Approximation, *RMSR* Root Mean Square Residual

**Fig. 1:**
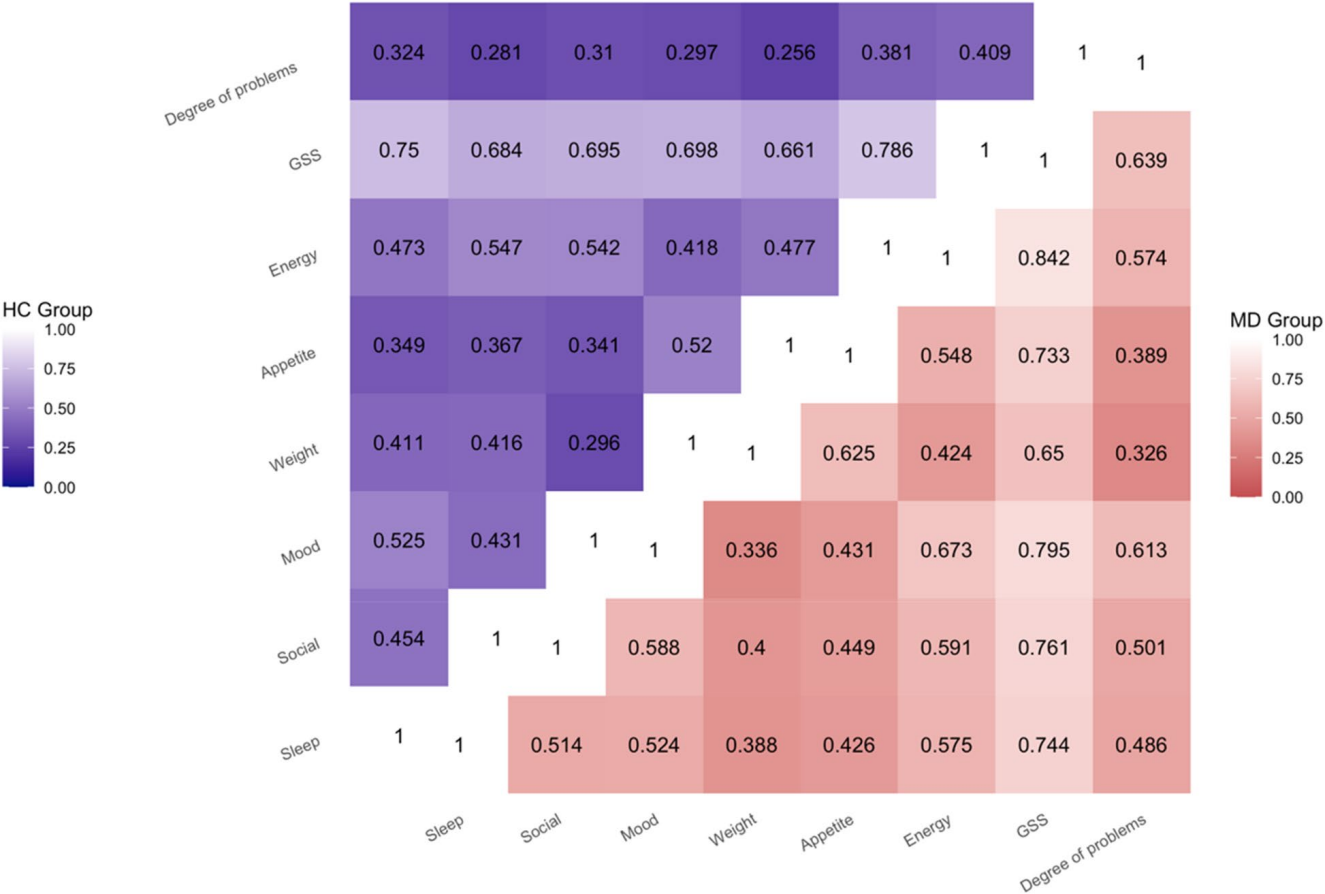
The correlation between the global seasonality score (GSS), six mood dimensions, and the degree of problems due to seasonal changes

Figure 1 illustrates the correlations between the GSS, six mood dimensions, and “the degree of problems due to seasonal changes”. The six symptom dimensions were significantly correlated with each other (*p* < 0.05), with correlation coefficients ranging from 0.34 to 0.67 in mood disorder patients and from 0.30 to 0.55 in the HC group. The GSS also showed a significant correlation with “the degree of problems due to seasonal changes”, with correlation coefficients of 0.64 for mood disorder patients and 0.41 for the HC group. Furthermore, “the degree of problems due to seasonal changes” was significantly correlated with each of the six symptom dimensions. In mood disorder patients, the correlation coefficients ranged from 0.33 to 0.61, while in the HC group, they ranged from 0.26 to 0.38.

### Assessment of seasonal patterns and inter-season consistency

**Fig. 2:**
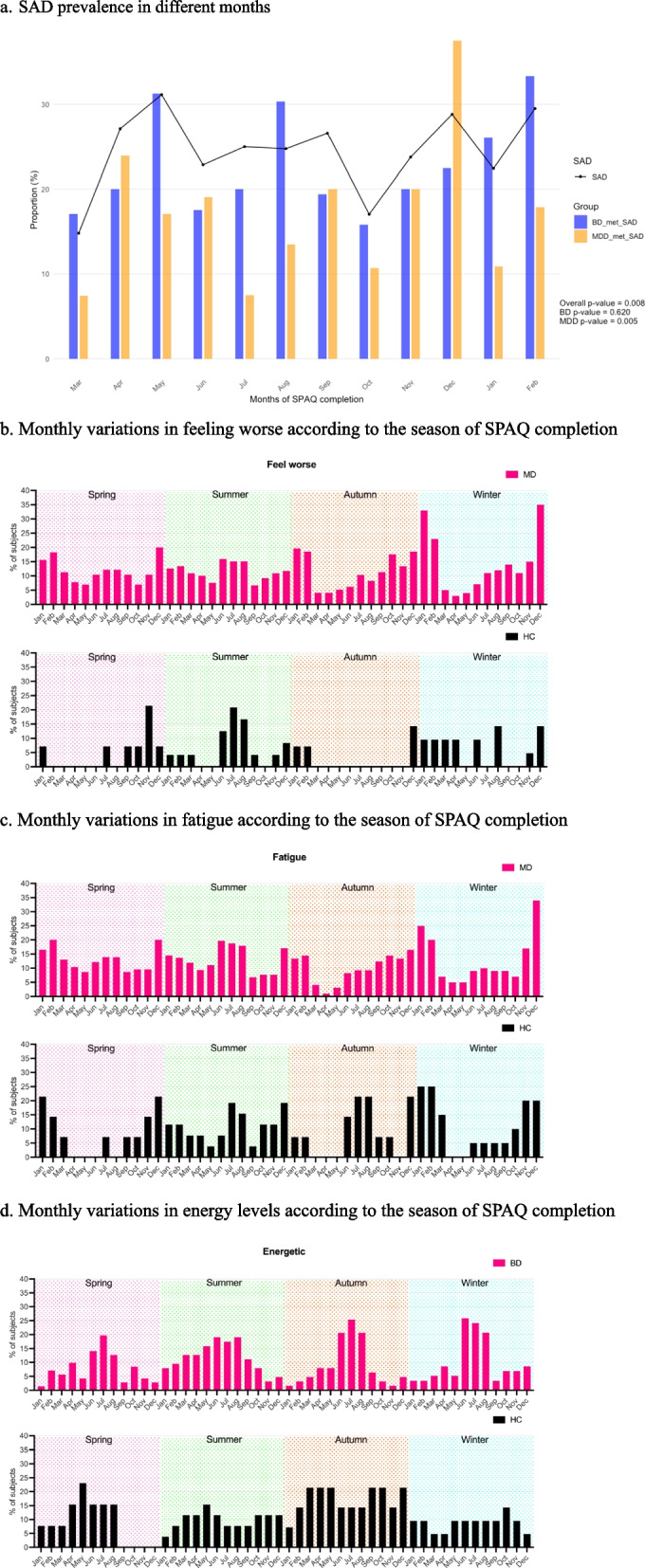
Variation of SAD prevalence, GSS, and symptom dimensions as the SPAQ is completed across different months and seasons. **a** SAD prevalence in different months. **b** Monthly variations in feeling worse as the SPAQ is completed across different seasons. **c** Monthly variations if fatigue as the SPAQ is completed across different seasons. **d** Monthly variations in energy levels as the SPAQ is completed across different seasons. Note: The monthly prevalence of SAD and the selected mood symptoms (feeling worse, fatigue, and energy) across the year based on responses of the seasonal pattern assessment questionnaire in every season

Figure 2 presents the variation in SAD prevalence and mood symptoms across different interview seasons. Among patients with BD, SAD prevalence ranged from 15.79% to 33.33%, while in those with MDD, it ranged from 7.41% to 37.50% (Fig. 2a). Seasonal differences in SAD prevalence were notable, with a maximum variation of 17.54% in BD and 30.09% in MDD, indicating substantial fluctuations across seasons. The monthly variation in SAD prevalence was significant in all the mood disorder samples (*p*-value = 0.008), with the peak observed in May (31.13%). In the BD group, higher SAD prevalence was observed in May (31.25%), August (30.30%), and February (33.33%), though the monthly variation was not significant (*p*-value = 0.62). Among patients with MDD, the variation in SAD prevalent across months was statistically significant (*p* = 0.005), with the highest percentage recorded in December (37.50%), followed by April (23.91%).

Figures 2b to d depict the monthly distribution of three mood symptoms among mood disorder patients and the HC group across distinct interview seasons. The prevalence of “feeling worst” and “fatigue” was higher during the winter months and remained consistent throughout all four interview seasons (Figs. 2b and c). Additionally, a peak prevalence was observed in the summer months during both the spring and summer interview seasons (Figs. 2b and c). When examining the “energetic” symptoms of BD patients, the highest prevalence was consistently observed during the summer months, regardless of the interview seasons (Fig. 2d). In the HC group, there was no evident pattern for “feeling worst” and “energetic” symptoms, while “fatigue” appeared to be consistently prevalent in the winter months across all four interview seasons.

In Table 4, we evaluated the consistency of participants’ responses to seasonal changes in six symptom dimensions across the four interview seasons. For mood disorder patients, the ICCR values for mood and energy level were 0.429 and 0.464, respectively. The GSS showed ICCR values of 0.520 for mood disorder patients. Additionally, the ICCR for the occurrence of manic symptoms in specific seasons was 0.437, while depressive symptoms had an ICCR of 0.316.

**Table 4:** Seasonal consistency in symptom dimensions, seasonality severity, and mood symptoms across four seasons in mood disorder patients (*n* = 121)

	BMS	WMS	k0	ICCR
*Symptom dimensions*				
Sleep	2.413	0.797	3.659	0.356
Social activity	2.237	0.931	3.651	0.278
Mood	3.095	0.825	3.659	0.429
Weight	2.083	0.741	3.659	0.331
Appetite	2.298	0.675	3.651	0.397
Energy level	2.971	0.714	3.659	0.464
*Seasonality severity*				
Global seasonality score	61.103	12.285	3.659	0.521
*Symptoms worsen in certain seasons*^a^				
Depressive symptoms	3.523	1.313	3.642	0.316
Manic symptoms	6.087	1.551	3.768	0.437

*BMS* Between mean square, *WMS* Within mean square, *ICCR* Intraclass correlation coefficient reliability

^a^This indicates that depressive or manic symptoms are more pronounced in specific seasons

## Discussion

The present study employed a combination of cross-sectional and longitudinal data to examine the response variation and validity of the SPAQ, a commonly used assessment tool in research on SAD. On average, 25.29% of mood disorder patients met the criteria for SAD, while an additional 13.57% met the criteria for S-SAD. These prevalence are lower than those reported in Western studies, where 30% to 40% of mood disorder patients met the criteria for SAD, and approximately 50% met the criteria for either S-SAD or SAD [19, 27]. Among patients with BD, our observed proportion of 25.29% is consistent with a previous meta-analysis that reported a proportion of approximately 27% based on either DSM-5 diagnosis or SPAQ screening [3]. When compared with other Asian countries, our results were similar to those reported in China (25%) and higher than in South Korea (17.5%) [11, 15].

Regarding the subtypes of seasonal patterns, the majority of SAD patients in our sample, regardless of mood disorder diagnosis, reported a winter pattern characterized by depressed mood in winter, with only a small group exhibiting a summer pattern. This is consistent with previous studies showing a predominance of winter patterns in depression admissions, although a minor summer peak has been observed in some populations [3, 28]. In contrast, manic episodes were more commonly associated with summer, supporting prior research that indicates a seasonal peak in mania during the summer months [3]. However, becasue Taiwan is located in a subtropical region – with less extreme photoperiod changes – the underlying mechanisms of SAD may differ from those in high-latitude countries [3, 14, 19]. Regional studies from Asia suggest that climatic and cultural factors may play important roles in shaping seasonal patterns. For instance, in India, heat has been implicated as a driver of summer-pattern depression, while manic episodes are more common during the rainy and winter seasons [16]. In Japan, sociocultural factors have been proposed as contributors to winter-pattern SAD [29]. In Taiwan, previous studies have shown that both sunshine duration and temperature influence seasonal mood changes, with different mood episode types showing distinct sensitivities to these climate factors [30].

In our study, an EFA with Promax rotation confirmed a two-factor structure for the six symptom dimensions among patients with mood disorder. This finding is consistent with previous research conducted by Mersch et al. [18] and Reynaud et al. [19]. One factor, referred to as the psychological factor, encompasses sleep, social activity, and energy level, while the other factor, known as the food-related factor, includes weight and appetite [18, 19]. Notably, we found that “sleep” loaded on both factors, deviating from previous studies where it was included soley in the food factor [18, 19, 31]. In the HC group, we identified a single factor that aligns with findings from Young et al. [20] in their study on healthy college students, with the ranking of factor loadings showing similar consistency.

Significant positive correlations were found between the GSS, six symptom dimensions, and the perception of “the degree of problems due to seasonal changes” in both the mood disorder and HC groups. These correlations ranged from moderate to strong, indicating a clear relationship between seasonal changes and mood experiences in both groups. Specifically, a moderate correlation (*r* = 0.54 – 0.67, *p* < 0.05) was observed between mood and energy level, which aligns with previous population-based surveys reporting correlations of *r* = 0.65—0.66 [19, 24]. However, our observed correlation was lower than that reported in a sample of individuals diagnosed with SAD using the DSM-5 criteria (*r* = 0.80) [18], suggesting that the relationship between mood and energy level may be stronger among those with clinically diagnosed SAD. Additionally, we found a moderate correlation between appetite and weight (*r* = 0.52—0.63, *p* < 0.05), as well as between appetite and energy level (*r* = 0.47 – 0.55, *p* < 0.05), which was consistent with previous studies [18, 19, 24]. Notably, the correlation between “the degree of problems due to seasonal changes” and the GSS was stronger in mood disorder patients (*r* = 0.64) compared to the HC group (*r* = 0.41) in the HC group, suggesting that higher GSS was associated with more severe seasonal disturbances. Furthermore, “the degree of problems due to seasonal changes” was also correlated with each of the six symptom dimensions, indicating that the severity of seasonal problems is linked to variations in individual symptoms across different dimensions.

The inter-season consistency of the SAD prevalence, GSS, and symptom dimensions suggests that while seasonal fluctuations are present, the overall variations remain modest. As shown in Fig. 2, the percentage of mood disorder patients meeting the criteria for SAD varied across months, ranging from 17.54% to 30.09% (Fig. 2a). While these differences suggest some level of seasonality, the relatively narrow range implies that the impact of seasonal changes on mood disturbances may not be as pronounced as previously thought. This is consistent with Lund and Hansen [32], who reported fluctuating SAD prevalence ranging from 5.6% to 14.4% over four months. Like their longitudinal design, our study followed participants over a year with assessments each season, allowing us to detect potential seasonal trends. However, as in Lund and Hansen’s study, we observed only modest fluctuations in SAD prevalence. A similar seasonal variation was found in GSS scores, with higher scores in winter and lower scores in summer [32], a trend also supported by findings in Norway [23]. When plotting the monthly prevalence of symptoms such as feeling worse, fatigue, and energy levels (Figs. 2b and d), we observed similar trends across the four seasons, regardless of when the questionnaire was administered. Symptoms of feeling worse and fatigue were more prevalent among mood disorder patients during winter, with another peak in the summer. These findings align with a recent study on depressed and bipolar patients, which reported similar seasonal symptom prevalence over a two-year period [19]. By re-testing participants each season, our study captures the stability of symptom patterns across the year, providing valuable insights into seasonal symptom fluctuations.

After analyzing the seasonal prevalence of symptoms, we examined the inter-season consistency of GSS, a key aim of our study. Among participants followed across four seasons using the SPAQ, we found acceptable consistency in GSS with an ICCR value of 0.521 for mood disorder patients and 0.449 for the HC group. This is the first evidence of the inter-seasonal characteristics of the Chinese version of GSS. Previous studies reported test–retest reliability values of 0.65 between summer and winter in healthy women [33] and 0.76 over two months in healthy individuals [20]. These results provide context for the consistency observed in our study and further validate the reliability of GSS across populations and time frames. When examining GSS dimensions, our results showed acceptable consistency (ICCR > 0.4) in mood and energy levels for both mood disorder patients and the HC group, while sleep and weight dimensions showed acceptable consistency only in the HC group. These findings align with research on college students, which also reported high test–retest reliability for mood and GSS over two seasons [34]. Our results extend this understanding across multiple seasons, further validating GSS’s robustness in capturing seasonal patterns in a clinical population. Despite demonstrating acceptable consistency, some fluctuations in GSS were observed across seasons. This suggests that GSS estimates may vary depending on when the questionnaire is completed, potentially influencing the measured prevalence of SAD. A possible explanation is that participants may have been influenced by their current symptoms at the time of assessment, affecting their ability to accurately evaluate their symptoms throughout the year [32].

One strength of this study is that it highlights the utility of the SPAQ not only as a research instrument but also as a practical screening tool in clinical settings. Clinicians can administer the SPAQ during the initial visit or routine appointments to identify those who may be vulnerable to seasonal mood fluctuations. The study’s results may help clinicians recognize high-risk periods and adjust treatment plans accordingly, such as altering medications or providing additional support to patients with recurrent seasonal patterns. Given its ease of use and demonstrated reliability across different seasons, the SPAQ shows promise as a valuable tool for monitoring seasonal trends and supporting individualized care in patients with mood disorder. However, the current study also has several limitations. Firstly, the sample size was limited, especially in the HC group, and therefore, a larger sample size is needed for more robust conclusions. However, it is worth noting that subjects with repeated measures, both mood disorder and HC, demonstrated high compliance. Secondly, the division of four seasons is based on meteorological seasons, which is commonly used in the Northern Hemisphere. However, this method may not fully represent the climate patterns of Taiwan, which is located in a subtropical region. In Taiwan, the Central Weather Bureau further distinguishes the spring rains, the plum rains, and the typhoon season within the four seasons. Despite this, for the ease of comparing research studies, we still prefer using the commonly used division in the Northern Hemisphere. Thirdly, there is a possibility of recall bias among subjects when answering the questionnaire. Although the majority of subjects were measured only once, it is important to note that 21.5% of subjects underwent prospective repeated measures. These repeated measures were not only to evaluate inter-season consistency but also indirectly suggest that the impact of recall bias is not significant. The results demonstrate acceptable reliability for the repeated measurements conducted over the four seasons. Fourth, it is worth noting that there was a two-thirds female representation in our sample. It is known that females have a higher risk for SAD [35]. The unequal gender distribution may lead to an overestimation of the proportions of SAD among mood disorder patients. To address this concern, we conducted a sensitivity analysis to estimate the proportion of SAD within both genders. The analysis revealed a proportion of 13.68% for males and 30.71% for females. Lastly, it should be considered that mood disorder patients had been on medication for a while, with most of them in remission. Over time, the seasonal patterns may subside. Nevertheless, the mood fluctuation in mood disorder still followed the seasonal changes, albeit without experiencing mood episodes, as humans are inherently influenced by seasonal variations [36]. We were able to measure the magnitude of these fluctuations using SPAQ. The results indicated that mood disorder patients remained more susceptible to seasonal variations compared to HC, which is reflected in the proportion of S-SAD. Overall, while the study has limitations, it provides valuable insights into the interplay between mood disorder, seasonal patterns, and the prevalence of SAD.

## Conclusions

In conclusion, this study provided insights into the structure of the Chinese version of SPAQ among mood disorder patients and the HC group in Taiwan, utilizing both a case–control design and a four-season follow-up approach. We also addressed concerns about the potential impact of season on the magnitude of GSS. Our findings shed light on the inter-season consistency of SPAQ, suggesting that it is a reliable tool for assessing seasonal patterns over time. This information is crucial for researchers and clinicians aiming to track and evaluate the seasonal variations in mood symptoms and related factors among individuals with mood disorder. The estimated proportion of SAD in our sample provides important local epidemiological data, offering insight into the burden of SAD in Taiwan’s subtropical climate. In particular, depressive episodes were more frequently reported during winter, whereas manic episodes peaked in summer, revealing a seasonal pattern of mood symptoms specific to this setting. These findings can help inform treatment strategies and enhance clinical awareness both in Taiwan and in regions with similar climate conditions. Overall, our study contributes to the knowledge base on the inter-season consistency of SPAQ and the proportion of SAD among mood disorder patients in subtropical regions. These findings have practical implications for the clinical assessment and management of seasonal recurrence in individuals with mood disorder.

## Supplementary Information

Supplementary Material 1.

## Data Availability

The dataset used in the current study is not publicly available due to participant privacy. However, data may be made available from the corresponding author upon reasonable request.

## Abbreviations

BD: Bipolar disorder
BD-I: Bipolar I disorder
BD-II: Bipolar II disorder
DSM-5: Diagnostic and Statistical Manual of Mental Disorders—fifth edition
EFA: Exploratory factor analysis
GSS: Global seasonality score
HC: Healthy controls
ICCR: Intraclass correlation coefficient reliability
MDD: Major depressive disorder
RMSEA: Root mean square error of approximation
RMSR: Root mean square residual
SAD: Seasonal affective disorder
SPAQ: Seasonal Pattern Assessment Questionnaire
S-SAD: Subsyndromal-SAD
