# Supplementary material for: Evaluation of seasonal variations for the seasonal pattern assessment in mood disorder patients and healthy controls
Source: BMC Psychiatry. 2025 May 7;25:458. doi: 10.1186/s12888-025-06916-y (PMC12057076; doi:10.1186/s12888-025-06916-y)
Supplement: Supplementary file 1 — Supplementary Material 1. [file 12888_2025_6916_MOESM1_ESM.docx]

**Supplementary Materials**

Table S1. distribution of Seasonal SPAQ completion and frequency of SAD criteria met

|  | n | percent (%) |
| --- | --- | --- |
| ***SPAQ completion by season*** | | |
| Spring | 294 | 25.52% |
| Summer | 287 | 24.91% |
| Autumn | 268 | 23.26% |
| Winter | 303 | 26.30% |
| ***Frequency of SAD criteria met*** | | |
| Once | 22 | 50.00% |
| Twice | 13 | 29.55% |
| Thrice | 4 | 9.09% |
| More than four times | 5 | 11.36% |
